# Changes of cerebral regional oxygen saturation during pneumoperitoneum and Trendelenburg position under propofol anesthesia: a prospective observational study

**DOI:** 10.1186/s12871-019-0736-4

**Published:** 2019-05-15

**Authors:** Toru Matsuoka, Tadahiko Ishiyama, Noriyuki Shintani, Masakazu Kotoda, Kazuha Mitsui, Takashi Matsukawa

**Affiliations:** 1 Department of Anesthesiology, Faculty of Medicine, University of Yamanashi, Chuo, Yamanashi Japan; 2 Surgical Center, University of Yamanashi Hospital, University of Yamanashi, 1110 Shimokato, Chuo, Yamanashi 409-3898 Japan

**Keywords:** Cerebral oxygenation, Endoscopic prostatic surgery, Pneumoperitoneum, Trendelenburg position

## Abstract

**Background:**

We evaluated the change of cerebral regional tissue oxygen saturation (rSO_2_) along with the pneumoperitoneum and the Trendelenburg position. We also assessed the relationship between the change of rSO_2_ and the changes of mean arterial blood pressure (MAP), heart rate (HR), arterial carbon dioxide tension (PaCO_2_), arterial oxygen tension (PaO_2_), or arterial oxygen saturation (SaO_2_).

**Methods:**

Forty-one adult patients who underwent a robotic assisted endoscopic prostatic surgery under propofol and remifentanil anesthesia were involved in this study. During the surgery, a pneumoperitoneum was established using carbon dioxide. Measurements of rSO_2_, MAP, HR, PaCO_2_, PaO_2_, and SaO_2_ were performed before the pneumoperitoneum (baseline), every 5 min after the onset of pneumoperitoneum, before the Trendelenburg position. After the onset of the Trendelenburg position, rSO_2_, MAP, HR were recorded at 5, 10, 20, 30, 45, and 60 min, and PaCO_2_, PaO_2_, and SaO_2_ were measured at 10, 30, and 60 min.

**Results:**

Before the pneumoperitoneum, left and right rSO_2_ were 67.9 ± 6.3% and 68.5 ± 7.0%. Ten minutes after the onset of pneumoperitoneum, significant increase in the rSO_2_ was observed (left: 69.6 ± 5.9%, right: 70.6 ± 7.4%). During the Trendelenburg position, the rSO_2_ increased initially and peaked at 5 min (left: 72.2 ± 6.5%, right: 73.1 ± 7.6%), then decreased. Multiple regression analysis showed that change of rSO_2_ correlated with MAP and PaCO_2_.

**Conclusions:**

Pneumoperitoneum and the Trendelenburg position in robotic-assisted endoscopic prostatic surgery did not worsen cerebral oxygenation. Arterial blood pressure is the critical factor in cerebral oxygenation.

**Trial registration:**

Japan Primary Registries Network (JPRN); UMIN-CTR ID; UMIN000026227 (retrospectively registered).

## Background

In robotic-assisted endoscopic prostatic surgery, a carbon dioxide pneumoperitoneum and the Trendelenburg position are essential. In the pneumoperitoneum and the Trendelenburg position, intracranial pressure was reported to increase [1]. In addition, pneumoperitoneum increases intraperitoneal pressure that leads to increase in intrathoracic pressure. Furthermore, the Trendelenburg position increases intrathoracic pressure. Increase in intrathoracic pressure should result in increase in central venous pressure [2]. Both intracranial pressure and central venous pressure increase in the pneumoperitoneum and the Trendelenburg position. The cerebral perfusion pressure is regarded as the mean arterial blood pressure (MAP) minus the intracranial pressure (when intracranial pressure > central venous pressure) or the central venous pressure (when central venous pressure > intracranial pressure) [3]. Therefore, unless the MAP changes the cerebral perfusion pressure decreased and cerebral circulation might be impaired in the pneumoperitoneum combined with the Trendelenburg position. Nevertheless, cerebral blood flow is maintained constantly within a wide range of cerebral perfusion pressure that is known as cerebral autoregulation [4]. On the other hand, cerebral blood flow has been reported to fluctuate even within autoregulation [5]. Therefore, cerebral blood flow may be unchanged or reduced after the pneumoperitoneum and the Trendelenburg position.

Measurement of cerebral regional tissue oxygen saturation values (rSO_2_) using near infrared spectroscopy can allow to assess cerebral circulation [6]. A previous study investigated that cerebral oxygenation during pneumoperitoneum in the Trendelenburg position [7]. However, the Trendelenburg position was firstly placed followed by pneumoperitoneum in that study. In robotic-assisted endoscopic prostatic surgery, pneumoperitoneum is performed before the Trendelenburg position.

We tested the following hypotheses. Firstly, rSO_2_ does not change after the pneumoperitoneum. Secondary, the Trendelenburg position combined with pneumoperitoneum does not alter rSO_2_. The primary outcome was the change of rSO_2_ along with the pneumoperitoneum and postural change. The secondary outcome was the relationship between the change of rSO_2_ and MAP, heart rate (HR), arterial carbon dioxide tension (PaCO_2_), arterial oxygen tension (PaO_2_), or arterial oxygen saturation (SaO_2_).

## Methods

This study was approved by the institutional review board of University of Yamanashi (study No. 488), and and was registered in the University Hospital Medical Information Network Clinical Trials Registry (UMIN-CTR) under study number UMIN000026227. Written informed consent was obtained from all patients.

## Anesthesia

Fifty-six adult patients (ASA physical status I or II) who underwent robotic-assisted endoscopic prostatic surgery were recruited. Patients having history of cerebral diseases such as cerebral infarction, cerebral hemorrhage, transient ischemic attack, or subarachnoid hemorrhage were excluded. No premedication was given. In the operating room, two sensors of near infrared spectroscopy (SAFB-SM, Covidien, Dublin, Ireland) were attached on the patient’s forehead to measure the left and right rSO_2_. A pulse oximeter was used to monitor percutaneous arterial oxygen saturation (SpO_2_). A bispectral index (BIS) sensor was also attached on the forehead (Model QUATRO, Covidien). An earphone-type infrared tympanic thermometer (CE Thermo, Nipro, Tokyo, Japan) was used to monitor body temperature. Non-invasive arterial blood pressure, HR, rSO_2_, SpO_2_, and body temperature were measured before induction of general anesthesia while the patient breathed room air (Pre values). Fluid was infused at 6 ml/kg/hr. Anesthesia was induced and maintained with propofol using target controlled infusion and remifentanil. Tracheal intubation was facilitated with rocuronium. After the induction of anesthesia, radial arterial catheter was placed to allow continuous monitoring of MAP and blood gas analysis (SaO_2_, PaO_2_, and PaCO_2_). The patient’s lungs were mechanically ventilated in a volume controlled mode (tidal volume: 6–8 ml/kg) with a positive end-expiratory pressure of 3 cm H_2_O. Peak airway pressure was controlled below 22 cmH_2_O. PaCO_2_ was maintaind between 35 and 45 mmHg. Fraction of inspired oxygen was adjusted to maintain PaO_2_ between 150 and 250 mmHg. Position of the blood pressure transducer was standardized to place at the level of the ear for every patient. Mean arterial blood pressure was controlled within 60–120 mmHg. If MAP fell below 60 mmHg, ephedrine or phenylephrine was given. If MAP went up over 120 mmHg, the infusion rate of remifentanil was increased. Bispectral index (BIS) was adjusted between 40 and 60 by controlling the target of propofol infusion. If rSO_2_ went down below 50%, or by 20% of the preanesthetic value, inspired oxygen was increased.

## Measurements

Before pneumoperitoneum, baseline measurements of rSO_2_, MAP, HR, SpO_2_, SaO_2_, PaO_2_, and PaCO_2_ were made. Pneumoperitoneum with intra-abdominal pressure of 10–15 mmHg was established and measurements were repeated every 5 min. Approximately 15 min after the establishment of the pneumoperitoneum, the Trendelenburg position with 30° head-down tilt was started. Before the start of the Trendelenburg position, rSO_2_, MAP, HR, SpO_2_, SaO_2_, PaO_2_, and PaCO_2_ were measured. Measurements of rSO_2_, MAP, HR, and SpO_2_ were made at 5, 10, 20, 30, 45, 60 min after the start of Trendelenburg position. After the onset of the Trendelenburg position, blood gas analysis was performed at 10, 30, 60 min. The measurements were not blinded to the anesthesiologists.

## Statistical analysis

We used Stat Flex version 6.0 (Artec, Osaka, Japan) for statistical analysis. Power analysis revealed that the sample size of 41 patients was sufficient to provided 80% power with an α level of 0.05 to detect mean differences of 5% in rSO_2_. Change in rSO_2_, MAP, HR, SpO_2_, SaO_2_, PaO_2_, and PaCO_2_ were examined via analysis of variance and Tukey post hoc comparisons. Multiple regression analysis was performed to estimate the relationship between rSO_2_ and MAP, HR, SpO_2_, SaO_2_, PaO_2_, or PaCO_2_. For multiple regression analysis, rSO_2_ data were averaged. When statistical significances were obtained after the multiple regression analysis, we performed linear regression analysis. Values are represented as means ± SDs; a *p* value < 0.05 was considered statistically significant.

## Results

**Fig. 1:**
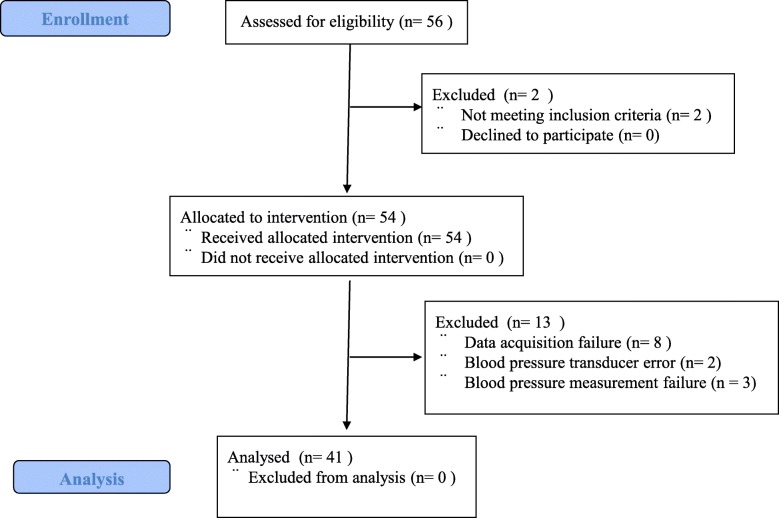
CONSORT diagram

Of 56 eligible patients, two patients failed to meet the inclusion criteria, and 13 patients developed the protocol fault such as data acquisition failure (*n* = 8), position of blood pressure transducer error (*n* = 2), and blood pressure measurement failure (bending of the arterial catheter) (*n* = 3). Therefore, we enrolled 41 patients (Fig. 1). Patients’ age, height, weight, body mass index (BMI) were 67 ± 6 yr., 164.8 ± 6.0 cm, 64.4 ± 9.2 kg, and 23.7 ± 2.9 kg/m^2^, respectively. One patient has BMI over 30 kg/m^2^ (30.6 kg/m^2^). No patients have comorbidities such as chronic obstructive pulmonary disease, heart failure, or uncontrolled hypertension. BIS before anesthesia was 93 ± 6. Propofol was infused at 2.8 ± 0.5 μg/ml and remifentanil was infused at 0.37 ± 0.10 μg/kg/min. BIS was maintained at 44 ± 6 during the study period. Body temperature before anesthesia was 36.3 ± 0.4 °C, and was maintained at 36.5 ± 0.5 °C during the study period.

**Fig. 2:**
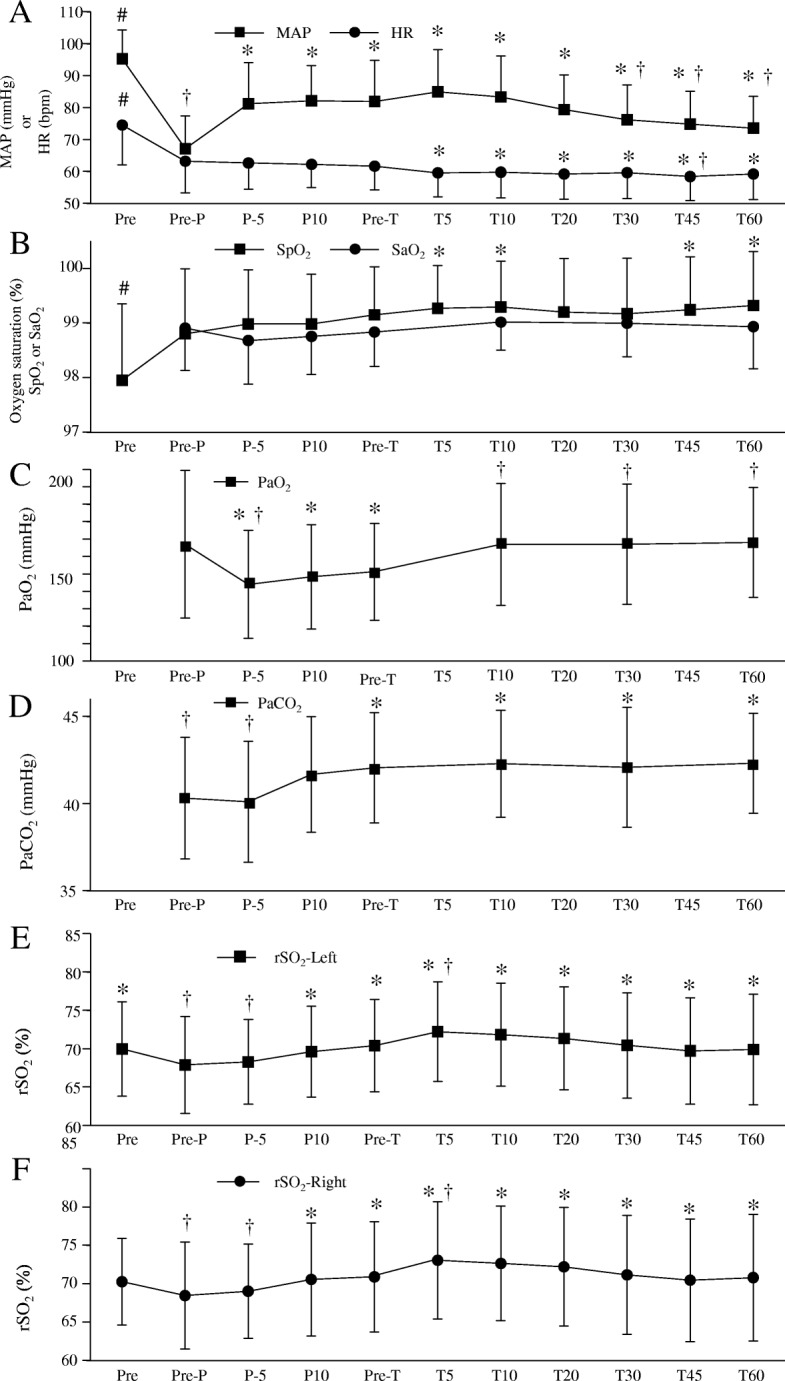
**a** Changes in mean arterial blood pressure (MAP) and heart rate (HR). **b** Changes in percutaneous (SpO_2_) and arterial (SaO_2_) oxygen saturation. **c** Changes in arterial oxygen tension (PaO_2_). **d** Changes in carbon dioxide tension (PaCO_2_). **e** Changes in left cerebral regional oxygen saturation (rSO_2_). **f** Changes in right cerebral regional oxygen saturation (rSO_2_). Pre: before the induction of anesthesia, Pre-P: just before the pneumoperitoneum, P-5, 10: 5, 10 min after the pneumoperitoneum, Pre-T: just before the Trendelenburg position (approximately 15 min after the pneumoperitoneum), T-5, 10, 20, 30, 45, 60: 5, 10, 20, 30, 45, 60 min after the Trendelenburg position. * *P* < 0.05, compared with Pre-P, † *p* < 0.05, compared with Pre-T, # *P* < 0.05, compared with other time points

Mean arterial blood pressure before anesthesia was 95 ± 9 mmHg. There were 8 patients who developed hypotension (MAP below 60 mmHg) after the induction of anesthesia. They were treated with ephedrine 5 mg and phenylephrine 0.05 mg. As shown in Fig. 2a, MAP decreased to 67 ± 10 mmHg before the pneumoperitoneum, and increased significantly after the pneumoperitoneum (81 ± 13 mmHg). After the Trendelenburg position, MAP slightly increased by 3 mmHg but the change was not statistically significant. Then it decreased significantly at 30, 45, and 60 min after the Trendelenburg position compared with that before the Trendelenburg position. Heart rate before anesthesia was 75 ± 12 beats/min. It decreased to 63 ± 10 beats/min before the pneumoperitoneum. Heart rate from 10 min to 60 min after the Trendelenburg position significantly decreased compared with that at before pneumoperitoneum (Fig. 2a). SpO_2_ before anesthesia (Pre) was 98.0 ± 1.4%, and increased to 99.0 ± 1.0% throughout the study period (Fig. 2b). SaO_2_ did not change in this study (Fig. 2b).

PaO_2_ before the pneumoperitoneum was 167.4 ± 43.9 mmHg. PaO_2_ decreased after the pneumoperitoneum, and increased after the Trendelenburg position. PaCO_2_ before the pneumoperitoneum was 40.3 ± 3.5 mmHg (Fig.2c). PaCO_2_ slightly but significantly increased after the pneumoperitoneum. PaCO_2_ remained high level during the pneumoperitoneum combined with the Trendelenburg position (Fig.2d).

Before general anesthesia, left (Fig.2e) and right (Fig.2f) rSO_2_ were 70.0 ± 6.2% and 70.3 ± 5.6%, respectively. Before the pneumoperitoneum, left and right rSO_2_ decreased to 67.9 ± 6.3% and 68.5 ± 7.0%. Ten min after the pneumoperitoneum, left and right rSO_2_ significantly increased to 69.6 ± 5.9% and 70.6 ± 7.4%. While patients were in the Trendelenburg position, left and right rSO_2_ significantly increased temporarily (5 min after the Trendelenburg position), and decreased to the baseline value afterwards but the change was not statistically significant.

Multiple regression analysis showed that change of rSO_2_ was correlated with MAP (*p* < 0.05) and PaCO_2_ (*p* < 0.0001) (Table 1). Linear regression analysis revealed that rSO_2_ = 65.717 + 0.0558 × MAP, r = 0.1141 (95% confidence interval; 0.0059–0.2197), and rSO_2_ = 45.3682 + 0.60127 × PaCO_2_, r = 0.3059 (95% confidence interval; 0.2037–0.4015).

**Table 1:** Multiple regression analysis between rSO_2_ and MAP, HR, SpO_2_, SaO_2_, PaO_2_, or PaCO_2_

	Unstandardized coefficients		Standardized coefficients	t	*p*-value
	B	SE	β		
	83.0782	70.0290			
MAP	0.07342	0.03057	0.1364	2.40144	0.0170
HR	0.01319	0.04505	0.0168	0.29271	0.7700
SpO_2_	−0.6075	0.55434	−0.0803	1.09593	0.2741
SaO_2_	0.13072	0.75372	0.0144	0.17343	0.8624
PaO_2_	0.01468	0.01322	0.0813	1.1022	0.2679
PaCO_2_	0.61094	0.11081	0.3222	5.51332	0.0000

Change of rSO_2_ was correlated with MAP (*p* < 0.05) and PaCO_2_ (*p* < 0.0001)

*rSO*_*2*_ cerebral regional tissue oxygen saturation, *MAP* mean arterial blood pressure, *HR* heart rate, *SpO*_*2*_ percutaneous arterial oxygen saturation, *SaO*_*2*_ arterial oxygen saturation, *B* regression coefficient, *SE* standard error, *β* standardized partial regression coefficient

## Discussion

We found in the present study that rSO_2_ increased after the pneumoperitoneum and further increased temporarily after the steep Trendelenburg position, and decreased afterwards. These changes were along with the alteration of MAP and PaCO_2_. However, the changes did not correlate with the changes of HR, PaO_2_, or SaO_2_.

The cerebral perfusion pressure is regarded as MAP minus central venous pressure (or intracranial pressure) [3]. A previous study reported that central venous pressure increased by 2–5 mmHg during the pneumoperitoneum [8, 9]. On the other hand, another previous study reported that MAP did not change after the pneumoperitoneum [9]. Thus, cerebral perfusion pressure should slightly decrease after the pneumoperitoneum. Contrary to the previous study [9], another study reported that pneumoperitoneum with a consequent increase in intracranial pressure produced systemic hypertension. [10]. Our study concurs with the latter study that MAP increased after the pneumoperitoneum. In patients in this study, cerebral autoregulation should be intact. Owing to the cerebral autoregulation, the cerebral blood flow is maintained constantly within a wide range of cerebral perfusion pressure. It is reasonable to assume that cerebral perfusion pressure remained normal level after the pneumoperitoneum. rSO_2_ reflects cerebral perfusion [11]. Therefore, we assumed that rSO_2_ would be unchanged after the pneumoperitoneum. However, rSO_2_ increased after the pneumoperitoneum in this study. In steady state, cerebral blood flow is maintained constant with static cerebral autoregulation [4]. In acute change in blood pressure, cerebral blood flow is compensatory adjusted by dynamic cerebral autoregulation [12, 13]. However, there is a time lag between the rise in blood pressure and the activation of dynamic cerebral autoregulation [14]. If blood pressure increased suddenly, cerebral blood flow may increase transiently. As a result, rSO_2_ increased.

After the Trendelenburg position combined with CO_2_ pneumoperitoneum, rSO_2_ increased initially. Some studies reported that central venous pressure increased by 10–16 mmHg during the Trendelenburg position combined with CO_2_ pneumoperitoneum [15–17]. On the other hand, blood pressure also increased by 10–15 mmHg during the Trendelenburg position combined with CO_2_ pneumoperitoneum in the previous studies [2, 15, 17]. In agreement with those studies, we observed that MAP increased by 16–18 mmHg at 5–10 min after the Trendelenburg position combined with CO_2_ pneumoperitoneum compared with that before the pneumoperitoneum. Change in cerebral perfusion pressure immediately after the Trendelenburg position was 16–18 mmHg (MAP change) minus 10–16 mmHg (assumed CVP change) [15–17]. The value was 0–8 mmHg. Whereas change in cerebral perfusion pressure after pneumoperitoneum was 14 mmHg (MAP change) minus 2–5 mmHg (assumed CVP change) [8, 9]. The value was 9–12 mmHg. Therefore, it is assumed that the cerebral perfusion pressure did not increase after the Trendelenburg position compared with that at the CO_2_ pneumoperitoneum. Cerebral perfusion pressure was not involved in the transient increase in rSO_2_ just after the Trendelenburg position combined with CO_2_ pneumoperitoneum. Schramm et al. reported that cerebral autoregulation deteriorated with Trendelenburg position combined with pneumoperitoneum [18]. Garrett et al. [19] reported that the cerebral blood flow velocity decreased when the posture was changed from the supine to the seated position. Postural change influences cerebral blood flow. Based on those previous studies, cerebral blood flow increased temporarily when the posture was changed from the supine to the Trendelenburg position. Due to transient increase in cerebral blood flow, rSO_2_ initially increased after the Trendelenburg position combined with CO_2_ pneumoperitoneum.

Cerebral blood flow varies with PaCO_2_. We recently reported that changes in rSO_2_ significantly correlated with changes in PaCO_2_. [20]. Abdominally insufflated carbon dioxide is absorbed into systemic circulation and is exhaled with ventilation. We attempted to adjust tidal volume and respiratory rate to maintain PaCO_2_ at 40 ± 5 mmHg. However, PaCO_2_ increased significantly after the pneumoperitoneum. Thus, rSO_2_ increased along with the rise in PaCO_2_. rSO_2_ gradually decreased after the pneumoperitoneum combined with the Trendelenburg position. Nevertheless, PaCO_2_ remained high level during the pneumoperitoneum combined with the Trendelenburg position. Although there was a correlation between rSO_2_ and PaCO_2_, PaCO_2_ may be less involved in the change of rSO_2_. On the other hand, MAP was observed highest at 5 min after the Trendelenburg position (T5 in the Fig. 2a) and it decreased thereafter. Head-down position in combination with a pneumoperitoneum impairs cerebral autoregulation over time [18]. It is likely that rSO_2_ changes with alterations in mean blood pressure rather than change of PaCO_2_.

Cerebral oxygenation can be monitored by rSO_2_ [21]. Cerebral oxygenation may influence the change of rSO_2_ in this study. According to the manufacturer, rSO_2_ reflects 25% arterial and 75% venous portion of blood. If cerebral oxygen consumption decreased, venous blood oxygen could be increased. As a result, rSO_2_ may have increased. However, BIS was unchanged after the induction of anesthesia. There were no factors that involved the decline in cerebral oxygen consumption after the pneumoperitoneum. Furthermore, PaO_2_ was significantly decreased after pneumoperitoneum. Therefore, oxygenation status was unlikely to participate in the rise of rSO_2_.

In this study, data before anesthesia were obtained under room air breathing and other data were measured under oxygen inspiration. Therefore, arterial oxygen saturation before anesthesia was significantly lower than that after the induction of anesthesia. However, rSO_2_ before anesthesia was significantly higher than that after the induction of anesthesia. Mean arterial blood pressure also declined after the induction of anesthesia. In addition, cerebral metabolic rate decreases after the induction of anesthesia that results in decrease in cerebral blood flow [22]. This phenomenon may indicate that rSO_2_ is firmly affected by MAP and cerebral blood flow rather than arterial oxygenation status in this study situation.

Cerebral autoregulation, which is expected to keep cerebral blood flow constant, mainly depends on cerebral perfusion pressure [4]. If cerebral perfusion pressure were too low (below the lower limit of autoregulation), cerebral blood flow might depend on MAP [23]. When the cerebral perfusion pressure was below the lower limit of autoregulation, cerebral blood flow should have been changed directly with the fluctuation of MAP. In this situation, rSO_2_ altered along with the change of MAP though decrease in blood pressure below the lower limit of autoregulation was not observed in this study. Nevertheless, individual variation in MAP while anesthetized and the lower limit of autoregulation might make the rSO_2_-MAP relationship different for different individuals.

In this study, PaO_2_ decreased after pneumoperitoneum. A previous study suggested that pneumoperitoneum elevated diaphragm that can lead to basilar atelectasis, with resulting right to left shunt formation [24]. Atelectasis caused by pneumoperitoneum may contribute to the decrease of PaO_2_. After the Trendelenburg position, PaO_2_ increased. Some studies reported that peak and mean airway pressure increased after the Trendelenburg position [25, 26]. Elevated airway pressure may have contributed to reduce atelectasis. As a result, PaO_2_ increased.

This study has some limitations. First, we did not measure central venous pressure because central venous catheterization is an invasive method and not always necessary for robotic-assisted endoscopic prostatic surgery. In many studies, central venous pressure was increased after pneumoperitoneum [8, 9] and the Trendelenburg position combined with pneumoperitoneum. [15–17] Therefore, the present study was based on an assumption that central venous pressure increased in this study. Second, cerebral blood flow was not measured. Transcranial Doppler can be utilized to assess the cerebral blood flow. However, due to the protection pads that supported the patient during the surgery, there was no space on the patient’s head for the Doppler probe attachment. Third, extracranial contamination affects rSO_2_. Davie et al. reported that the extracranial contamination potentially affected rSO_2_ [27]. They also indicated that forehead skin blood made an impact on rSO_2_ [27]. Trendelenburg position may cause venous stasis, which could result in the increase of venous portion of blood. Therefore, Trendelenburg position may have affected the relative arterial and venous content of the forehead skin blood. Extracranial contamination might have influenced rSO_2_ in this study.

## Conclusions

In conclusion, pneumoperitoneum and the Trendelenburg position in robotic-assisted endoscopic prostatic surgery did not aggravate cerebral oxygenation. Changes in rSO2 were associated with the alteration of MAP and PaCO2, but did not correlate with the changes of HR, PaO2, or SaO2, indicating that arterial blood pressure is the critical factor in the cerebral oxygenation.

## Abbreviations

BIS: Bispectral index
HR: Heart rate
MAP: Mean arterial blood pressure
PaCO_2_: Arterial carbon dioxide tension
PaO_2_: Arterial oxygen tension
rSO_2_: Cerebral regional tissue oxygen saturation
SaO_2_: Arterial oxygen saturation
SpO_2_: Percutaneous arterial oxygen saturation

## References

[1] Halverson A, Buchanan R, Jacobs L, Shayani V, Hunt T, Riedel C (1998). Evaluation of mechanism of increased intracranial pressure with insufflation. Surg Endosc.

[2] Kalmar AF, Foubert L, Hendrickx JF, Mottrie A, Absalom A, Mortier EP (2010). Influence of steep Trendelenburg position and CO_2_ pneumoperitoneum on cardiovascular, cerebrovascular, and respiratory homeostasis during robotic prostatectomy. Br J Anaesth.

[3] Munis JR, Lozada LJ (2000). Giraffes, siphons, and starling resistors. Cerebral perfusion pressure revisited. J Neurosurg Anesthesiol.

[4] Paulson OB, Strandgaard S, Edvinsson L (1990). Cerebral autoregulation. Cerebrovasc Brain Metab Rev.

[5] Tzeng YC, Ainslie PN (2014). Blood pressure regulation IX: cerebral autoregulation under blood pressure challenges. Eur J Appl Physiol.

[6] Ali AM, Green D, Zayed H, Halawa M, El-Sakka K, Rashid HI (2011). Cerebral monitoring in patients undergoing carotid endarterectomy using a triple assessment technique. Interact Cardiovasc Thorac Surg.

[7] Park EY, Koo BN, Min KT, Nam SH (2009). The effect of pneumoperitoneum in the steep Trendelenburg position on cerebral oxygenation. Acta Anaesthesiol Scand.

[8] Andersson L, Wallin CJ, Sollevi A, Odeberg-Wernerman S (1999). Pneumoperitoneum in healthy humans does not affect central blood volume or cardiac output. Acta Anaesthesiol Scand.

[9] Hänel F, Blobner M, Bogdanski R, Werner C (2001). Effects of carbon dioxide pneumoperitoneum on cerebral hemodynamics in pigs. J Neurosurg Anesthesiol.

[10] Ben-Haim M, Mandeli J, Friedman RL, Rosenthal RJ (2000). Mechanisms of systemic hypertension during acute elevation of intraabdominal pressure. J Surg Res.

[11] Rigamonti A, Scandroglio M, Minicucci F, Magrin S, Carozzo A, Casati A (2005). A clinical evaluation of near-infrared cerebral oximetry in the awake patient to monitor cerebral perfusion during carotid endarterectomy. J Clin Anesth.

[12] Kurazumi T, Ogawa Y, Yanagida R, Morisaki H, Iwasaki KI (2017). Dynamic cerebral autoregulation during the combination of mild hypercapnia and cephalad fluid shift. Aerosp Med Hum Perform.

[13] Zhang R, Zuckerman JH, Giller CA, Levine BD (1998). Transfer function analysis of dynamic cerebral autoregulation in humans. Am J Phys.

[14] Chiu CC, Yeh SJ (2001). Assessment of cerebral autoregulation using time-domain cross-correlation analysis. Comput Biol Med.

[15] Choi SH, Lee SJ, Rha KH, Shin SK, Oh YJ (2008). The effect of pneumoperitoneum and Trendelenburg position on acute cerebral blood flow-carbon dioxide reactivity under sevoflurane anaesthesia. Anaesthesia.

[16] Doe A, Kumagai M, Tamura Y, Sakai A, Suzuki K (2016). A comparative analysis of the effects of sevoflurane and propofol on cerebral oxygenation during steep Trendelenburg position and pneumoperitoneum for robotic-assisted laparoscopic prostatectomy. J Anesth.

[17] Kalmar AF, Dewaele F, Foubert L, Hendrickx JF, Heeremans EH, Struys MM (2012). Cerebral haemodynamic physiology during steep Trendelenburg position and CO_2_ pneumoperitoneum. Br J Anaesth.

[18] Schramm P, Treiber AH, Berres M, Pestel G, Engelhard K, Werner C (2014). Time course of cerebrovascular autoregulation during extreme Trendelenburg position for robotic-assisted prostatic surgery. Anaesthesia.

[19] Garrett Zachary K., Pearson James, Subudhi Andrew W. (2017). Postural effects on cerebral blood flow and autoregulation. Physiological Reports.

[20] Ishiyama T, Kotoda M, Asano N, Ikemoto K, Shintani N, Matsuoka T (2016). Effects of hyperventilation on cerebral oxygen saturation estimated using near-infrared spectroscopy: a randomised comparison between propofol and sevoflurane anaesthesia. Eur J Anaesthesiol.

[21] Aliane J, Duale C, Guesmi N, Baud C, Rosset E, Pereira B (2017). Compared effects on cerebral oxygenation of ephedrine vs phenylephrine to treat hypotension during carotid endarterectomy. Clin Exp Pharmacol Physiol.

[22] Conti A, Iacopino DG, Fodale V, Micalizzi S, Penna O, Santamaria LB (2006). Cerebral haemodynamic changes during propofol-remifentanil or sevoflurane anaesthesia: transcranial Doppler study under bispectral index monitoring. Br J Anaesth.

[23] Pater PM, Drummond JC, Miller RD (2010). Cerebral physiology and the effects of anesthetic drugs. Miller’s Anesthesia.

[24] Haydon GH, Dillon J, Simpson KJ, Thomas H, Hayes PC (1996). Hypoxemia during diagnostic laparoscopy: a prospective study. Gastrointest Endosc.

[25] Assad OM, El Sayed AA, Khalil MA (2016). Comparison of volume-controlled ventilation and pressure-controlled ventilation volume guaranteed during laparoscopic surgery in Trendelenburg position. J Clin Anesth.

[26] Kim MS, Soh S, Kim SY, Song MS, Park JH (2018). Comparisons of pressure-controlled ventilation with volume guarantee and volume-controlled 1:1 equal ratio ventilation on oxygenation and respiratory mechanics during robot-assisted laparoscopic radical prostatectomy: a randomized-controlled trial. Int J Med Sci.

[27] Davie SN, Grocott HP (2012). Impact of extracranial contamination on regional cerebral oxygen saturation: a comparison of three cerebral oximetry technologies. Anesthesiology.

